# Expression profiles of host miRNAs and circRNAs and ceRNA network during *Toxoplasma gondii* lytic cycle

**DOI:** 10.1007/s00436-024-08152-x

**Published:** 2024-02-29

**Authors:** Sha-Sha Wang, Xiangwei Wang, Jun-Jun He, Wen-Bin Zheng, Xing-Quan Zhu, Hany M. Elsheikha, Chun-Xue Zhou

**Affiliations:** 1 State Key Laboratory for Animal Disease Control and Prevention, College of Veterinary Medicine, Lanzhou University, Lanzhou Veterinary Research Institute, Chinese Academy of Agricultural Sciences, Lanzhou, 730000 Gansu Province China; 2 Key Laboratory of Veterinary Public Health of Higher Education of Yunnan Province, College of Veterinary Medicine, Yunnan Agricultural University, Kunming, 650500 Yunnan Province China; 3 College of Veterinary Medicine, Shanxi Agricultural University, Taigu, 030801 China; 4 Faculty of Medicine and Health Sciences, School of Veterinary Medicine and Science, University of Nottingham, Sutton Bonington Campus, Loughborough, LE12 5RD UK; 5 Department of Pathogen Biology, School of Basic Medical Sciences, Cheeloo College of Medicine, Shandong University, Jinan, 250100 Shandong Province China

**Keywords:** *Toxoplasma gondii*, RNA-seq, circRNA, miRNA, Immune response, Host–pathogen interaction

## Abstract

*Toxoplasma gondii* is an opportunistic protozoan parasite that is highly prevalent in the human population and can lead to adverse health consequences in immunocompromised patients and pregnant women. Noncoding RNAs, such as microRNAs (miRNAs) and circular RNAs (circRNAs), play important regulatory roles in the pathogenesis of many infections. However, the differentially expressed (DE) miRNAs and circRNAs implicated in the host cell response during the lytic cycle of *T. gondii* are unknown. In this study, we profiled the expression of miRNAs and circRNAs in human foreskin fibroblasts (HFFs) at different time points after *T. gondii* infection using RNA sequencing (RNA-seq). We identified a total of 7, 7, 27, 45, 70, 148, 203, and 217 DEmiRNAs and 276, 355, 782, 1863, 1738, 6336, 1229, and 1680 DEcircRNAs at 1.5, 3, 6, 9, 12, 24, 36, and 48 h post infection (hpi), respectively. Gene Ontology and Kyoto Encyclopedia of Genes and Genomes pathway enrichment analyses revealed that the DE transcripts were enriched in immune response, apoptosis, signal transduction, and metabolism-related pathways. These findings provide new insight into the involvement of miRNAs and circRNAs in the host response to *T. gondii* infection.

**Supplementary Information:**

The online version contains supplementary material available at 10.1007/s00436-024-08152-x.

## Introduction

Toxoplasmosis is caused by *Toxoplasma gondii*, an apicomplexan protozoan parasite infecting approximately one-third of the world’s human population (Hill and Dubey 2002). Human infection mainly occurs via ingesting food or water contaminated with the parasite cyst or oocyst stage. Healthy people usually exhibit no clinical symptoms in response to *T. gondii* infection. However, individuals with immune deficiency, such as those with AIDS, can exhibit serious symptoms, such as headache and seizures (Jones-Brando et al. 2003; Elsheikha et al. 2020). Following the initial infection, *T. gondii* establishes a lifelong latent infection which can be reactivated in the immunocompromised hosts (Elsheikha et al. 2020). Pregnant women can transmit this parasite to their unborn offspring, which may lead to abortion or stillbirth (Elsheikha 2008; Pappas et al. 2009). Given the limitations of the commercially available medications and the lack of a vaccine to prevent infection in humans (Wang et al. 2019), there is a pressing need to improve the understanding of the interaction between *T. gondii* and its hosts and elucidate the precise mechanisms underlying *T. gondii* pathogenesis during *T. gondii* lytic cycle (Black and Boothroyd 2000).

To address the current unmet needs and establish a pipeline of new innovative treatment solutions for effective control of *T. gondii* infection, it is crucial to identify the molecules that underpin the host response to *T. gondii* infection, since these molecules may serve as therapeutic targets. Previous analysis using global transcriptomics (Jia et al. 2013; Tanaka et al. 2013; Radke et al. 2018; Wang et al. 2022; Hu et al. 2020; Xie et al. 2022) and proteomics (Sahu et al. 2014; Lv et al. 2017; Yang et al. 2018; Zhou et al. 2021) revealed some mechanisms by which *T. gondii* can alter gene expression and protein abundance in various tissues in the affected hosts. *T. gondii* infection can also alter the expression of non-coding RNA in vivo and in vitro (Hunter and Sibley 2012; Liu et al. 2018; Wang et al. 2018). This parasite hijacks host gene expression and modulates immune response pathways, including those involved in apoptosis and cytokine production (Judice et al. 2016). Despite the significant progress that has been achieved in understanding the relationship between *T. gondii* infection and host cells, the functional involvement of certain molecules, such as microRNAs (miRNAs) and circular RNAs (circRNAs), in the parasite pathogenesis and their contribution to anti-*T. gondii* immune response remain largely unexplored. Better understanding of the function of miRNAs and circRNAs implicated in *T. gondii* infection can reveal new insights into the interaction between the host and *T. gondii*.

microRNAs and circular RNAs play a vital role in post-transcriptional regulation of gene expression and protein activity (Zhang et al. 2022). circRNAs are a subclass of non-coding RNAs involved in the regulation of many biological processes, such as regulation of immune responses. They can compete with and influence the ability of miRNAs to regulate gene expression through a sponge-like mechanism (Granados-Riveron and Aquino-Jarquin 2016). Various circRNAs are involved in regulating or modulating viral infection and antiviral innate immunity (Li et al. 2017; Lu et al. 2020). Additionally, miRNAs and circRNAs can act as regulatory factors in the pathogenesis of neurological diseases, such as epilepsy, Parkinson’s disease (PD), and Alzheimer’s disease (AD) (Shao and Chen 2016; Awuson-David et al. 2023; Dong et al. 2023). *T. gondii* infection also alters the expression of host miRNAs and circRNAs in vivo to promote parasite replication (Xu et al. 2013; He et al. 2016; Cong et al. 2017; Hu et al. 2018; Zou et al. 2022).

Host-parasite interactions operate at the transcriptional and epigenetic levels and are critical for establishing a latent or lytic *T. gondii* infection. However, the functional involvement of miRNAs and circRNAs in the immunity and pathogenesis of *T. gondii* lytic cycle remains largely unknown. In the present study, we employed RNA sequencing (RNA-seq) analysis to investigate the changes in the expression of miRNAs and circRNAs in human foreskin fibroblasts (HFFs) during *T. gondii* infection lytic cycle. Another objective of the study was to identify the differentially expressed (DE) miRNAs and circRNAs and predict the miRNA-circRNA interactions. Our data provides new insight into the roles of miRNAs and circRNAs in regulating anti-*T. gondii* immunity.

## Materials and methods

### Parasites and cell culture

The HFF cell line was cultured in Dulbecco’s modified Eagle’s medium (DMEM) containing 10% (v/v) fetal bovine serum, 100 U/mL penicillin, and 100 U/mL streptomycin and maintained in an incubator at 37 °C and 5% CO_2_. Tachyzoites of *T. gondii* RH strain were maintained in HFF monolayers as previously described (Zhou et al. 2016).

### *T. gondii* infection and sample collection

Monolayers of HFF cells, at 80% confluence in 25-cm^2^ tissue culture flasks, were infected by *T. gondii* RH strain at a multiplicity of infection (MOI) of 5. The mock-infected (control) HFFs were maintained under the same culture conditions except that cells were not infected by *T. gondii*. The cell samples were collected at 0, 1.5, 3, 6, 9, 12, 24, 36, and 48 h post infection (hpi). The RNA-seq samples were composed of 3 separate biological replicates for each time point. The collected cell samples were stored at − 80 °C until use.

### RNA extraction

Total RNA was extracted using Trizol method (Invitrogen, Carlsbad, CA, USA) according to the manufacturer’s instructions. The quantity and quality of the extracted RNA were determined by using the NanoDrop spectrophotometer and Agilent 2100 Bioanalyzer (Thermo Fisher Scientific, MA, USA), respectively.

### RNA-seq

The RNA integrity number (RIN) was used to evaluate the quality and integrity of the isolated RNA and only samples with RIN ≥ 8.0 were used for RNA-seq. The construction of the sequencing libraries and sequencing were performed at Beijing Genomics Institute in Shenzhen. Briefly, small RNA sequencing libraries were constructed and 50 bp single-end reads were generated using HiSeq 2500 sequencing platform. Raw data of fastq format were firstly processed using in-house perlscripts. Q20, Q30, and GC-content of the clean data were calculated. To analyze the small RNA expression and distribution on the genome, small RNA tags were mapped against the reference sequence by using Bowtie (Langmead et al. 2009). For the construction of circRNA library, 5 μg RNA per sample was used as input material. Ribosomal RNA was removed by Epicentre Ribozero™ rRNA Removal Kit (Epicentre, USA) followed by the linear RNA digestion with RNase R (Epicentre, USA). The sequencing libraries were constructed by NEBNext® Ultra™ Directional RNA Library Prep Kit for Illumina® (NEB, USA) and 150 bp paired-end reads were generated by Illumina HiSeq 4000 sequencing platform. The reference *Homo sapiens* genome was downloaded from the NCBI genome website. Then, index of the *H. sapiens* genome was built using Bowtie2 v2.2.8 and paired-end clean reads were aligned to the reference genome (Langmead et al. 2009).

### Data processing and differential expression profiling

The raw sequencing data was filtered with SOAPnuke (v1.5.2) (Kim et al. 2015) to obtain high-quality reads. HISAT2 was used to align the clean reads to the *Homo sapiens* reference genome (GRCh38.p14) in the NCBI (Langmead and Salzberg 2012), followed by the application of Bowtie2 (v2.2.5) (Li and Dewey 2011) to align the clean reads to database, novel, coding, and noncoding transcripts, established by Beijing Genomic Institute (BGI, Shenzhen, China). RSEM (v1.2.12) was used to calculate the gene expression levels (Love et al. 2014), and differential expression analysis was performed using DESeq2 R package (v1.4.5) with *Q* value ≤ 0.05 (Abdi 2007). The Venn diagrams were constructed using the VennDiagram R package (v1.7.3). The hierarchical clustering using heatmaps were generated using the ComplexHeatmap R package (v2.16.0).

### Prediction of miRNAs and circRNAs

The data of miRBase20.0 were used as miRNA reference and the modified software miRDeep2 (Friedländer et al. 2012) was used to identify the known miRNAs. The software miREvo (Wen et al. 2012) and miRDeep2 (Friedländer et al. 2012) were combined to predict novel miRNA by exploring the secondary structure, the Dicer cleavage site, and the minimum free energy of the small RNA tags unannotated in the former steps. CircRNAs were detected and identified using the find_circ script (Memczak et al. 2013) and CIRI2 tool (Gao et al. 2018), and circRNA sequences were predicted based on junction reads and GT-AG cleavage signals (Gao et al. 2015). The miRNA target sites in the circRNAs were predicted using miRanda (version 3.3a) (Enright et al. 2003). The miRanda, PITA, and RNAhybrid softwares were used to predict potential target genes of the differentially expressed miRNAs.

### Gene ontology and KEGG enrichment analysis

The DEmiRNAs and DEcircRNAs host genes were subjected to Gene Ontology (GO) enrichment analysis using web-based GO software (http:/www.geneontology.org) and Kyoto Encyclopedia of Genes and Genomes (KEGG) pathway analysis using KEGG database (http://www.genome.jp/kegg/). Significant levels of enriched GO terms and pathways were corrected by *Q* value with a threshold (*Q* value ≤ 0.05) by Bonferroni method (Hu et al. 2020). Sankey diagrams were constructed using the ggsankey R package to display the common GO terms and KEGG pathways at different time points after infection.

### Quantitative real-time PCR analysis

To validate the expression of miRNAs and circRNAs obtained by RNA-seq, quantitative real-time PCR (qRT-PCR) was performed on a LightCycler 480 (Roche, Basel, Switzerland) using TB Green® Premix Ex Taq™ II (Tli RNaseH Plus) (Takara, Shuzo, Kyoto, Japan). The reactions were performed at 95 °C for 30 s, followed by 40 cycles at 95 °C for 5 s and 60 °C for 30 s. Three replicate reactions were performed for each gene, and the glyceraldehyde-3-phosphate dehydrogenase (*GAPDH*) gene and *U6* gene were included as internal controls for circRNA and miRNA, respectively. The 2^−∆∆CT^ method was used to calculate gene expression. The primer sequences used in this study are listed in Table 1.

**Table 1:** qRT-PCR primers used in this study

Target gene	Forward primer (5′-3′)	Reverse primers (5′-3′)
hsa-miR-663b	GATAAGGTGGCCCGGCC	AGTGCAGGGTCCGAGGTATT
hsa-miR-671-5p	AGGAAGCCCTGGAGGGG	AGTGCAGGGTCCGAGGTATT
hsa-miR-1246	GCGCGAATGGATTTTTGG	AGTGCAGGGTCCGAGGTATT
hsa_circ_0083081	GCCTCGTGCTCTTCTCGGTTG	CGGAGGAAAAGATGGTGGCGATC
hsa_circ_0133744	CCTATCTTTGGGCCTTTGGTGGAC	AGAAACTGCGTGATCAGCGTAGC
hsa_circ_0083083	CCGTGGCAGTGATGGAAGTGTG	GCAGGACCAGCGTTACCAACAG
GAPDH*	CACCACACCTTCTACAAC	TCTGGGTCATCTTCTCAC
U6*	CTCGCTTCGGCAGCACA	AACGCTTCACGAATTTGCGT

^*^GADPH and *U6* genes were used for cirRNA and miRNA normalization, respectively

### Prediction of circRNA-miRNA interactions

We constructed the circRNA-miRNA interaction network using miRanda software, based on the miRNA binding sites on circRNAs and the targeted relationship of DEcircRNAs and their downstream target miRNAs, as previously described (Enright et al. 2003). The competing endogenous RNA (ceRNA) network was visualized based on the potential target relationships between DEmiRNAs and DEcircRNAs using Cytoscape v3.6.0.

### Statistical analysis

All statistical analyses were performed using GraphPad Prism version 7.0 (GraphPad Software Inc, San Diego, CA, USA). All data represent the mean ± standard deviation of triplicate. A *p* value of < 0.05 was considered statistically significant.

## Results

### Identification of the differentially expressed miRNAs

**Fig. 1:**
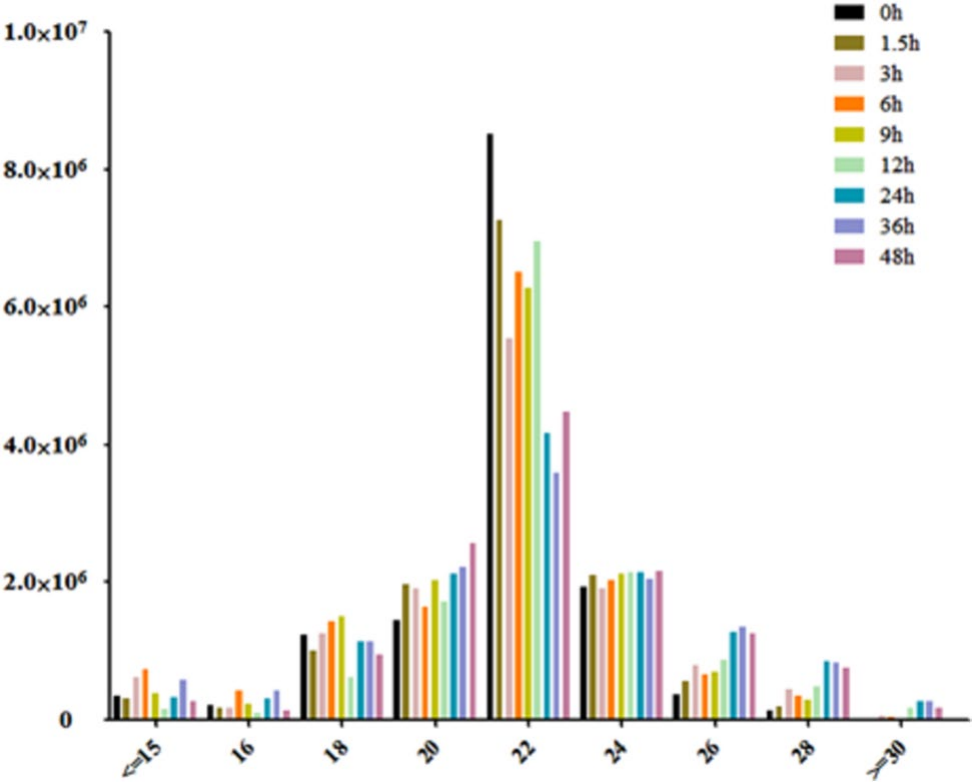
Basic information of microRNAs (miRNAs) showing miRNA length distribution (x-axis) and the frequency of miRNA of that length (y-axis) identified at different time points after *T. gondii* infection

**Fig. 2:**
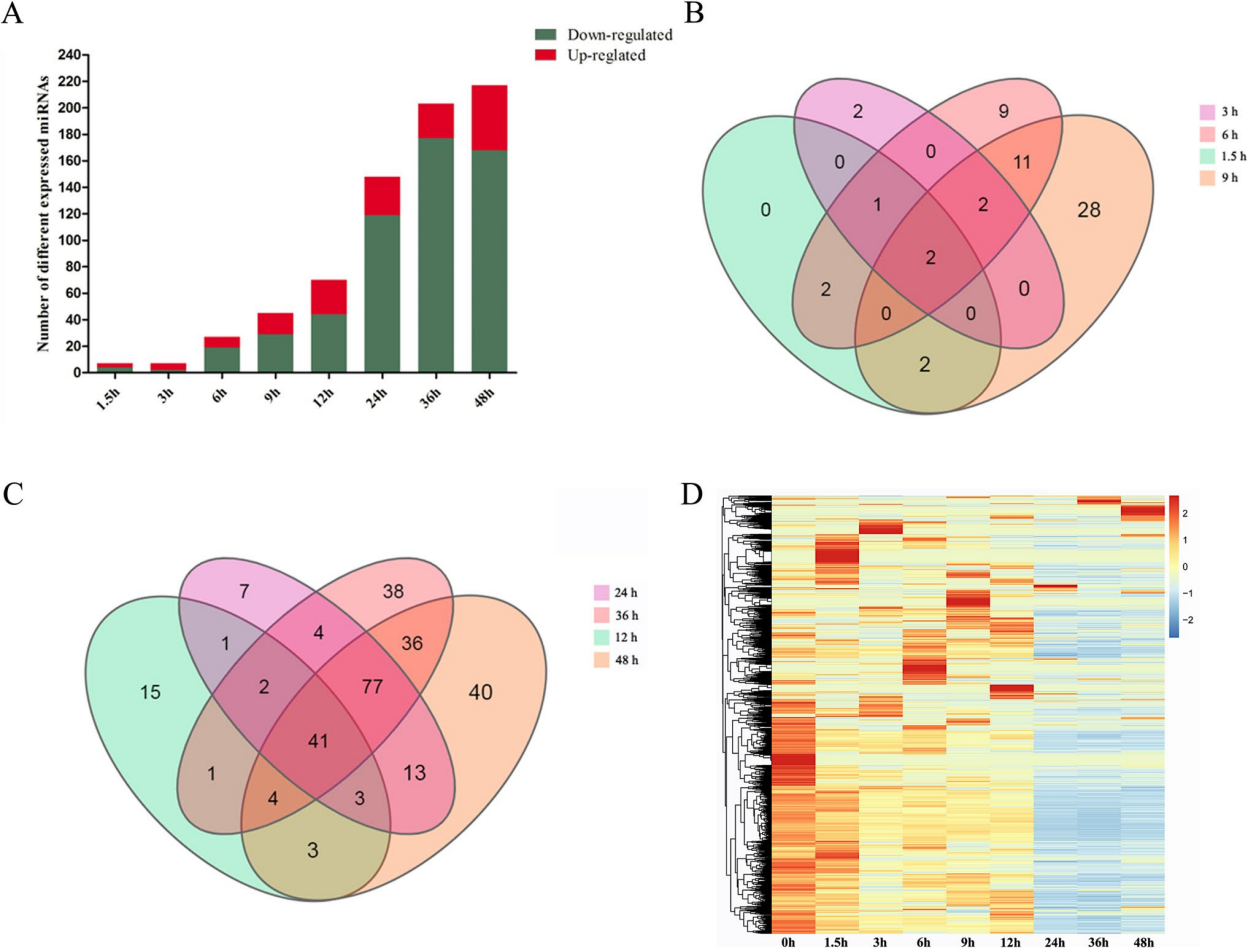
Global changes in miRNA expression after *T. gondii* infection. (**A**) DEmiRNAs detected in HFF cells at different time points after *T. gondii* infection. The x-axis shows the time points after *T. gondii* infection and the y-axis shows the number of DEmiRNAs. Red and green colors represent the upregulated and downregulated DEmiRNAs, respectively. Four-ellipse Venn diagram outlining the common and unique DEmiRNAs at 1.5 to 9 hpi (**B**) and 12 to 48 hpi (**C**). Hierarchical clustering heatmap of the DEmiRNAs at different time points after *T. gondii* infection. Clustering was performed as log_10_ (FPKM + 1) values, and red color indicates highly expressed genes and blue color indicates low expressed genes (**D**). The values of miRNA expression are indicated by the color scale; from red to blue indicating highly expressed and low expressed miRNAs, respectively. Each column represents a time point after infection, and each row represents one miRNA

To investigate the dynamic expression and function of miRNAs in the lytic cycle of *T. gondii* infection, samples were collected for transcriptome sequencing at 0, 1.5, 3, 6, 9, 12, 24, 36, and 48 hpi.* Q* value < 0.05 and |log_2_ Fold change|> 1 were used as thresholds to identify the DE genes. As shown in Fig. 1, the dominant small RNAs (sRNAs) were 20–24 nt in length, and most sRNAs were 22 nt, which is consistent with the typical size of miRNA. Expression analysis identified 7 DEmiRNAs (4 upregulated and 3 downregulated), 7 DEmiRNAs (2 upregulated and 5 downregulated), 27 DEmiRNAs (19 upregulated and 8 downregulated), 45 DEmiRNAs (29 upregulated and 16 downregulated), 70 DEmiRNAs (44 upregulated and 26 downregulated), 148 DEmiRNAs (119 upregulated and 29 downregulated), 203 DEmiRNAs (177 upregulated and 26 downregulated), and 217 DEmiRNAs (168 upregulated and 49 downregulated) at 1.5, 3, 6, 9, 12, 24, 36, and 48 hpi, respectively (Fig. 2A). Two DEmiRNAs (hsa-miR-1246 and hsa-miR-663b) were commonly expressed during 1.5 to 9 hpi (Fig. 2B). Additionally, 41 DEmiRNAs were co-expressed during 12 to 48 hpi (Fig. 2C). The hierarchical clustering of miRNA expression profiles between control and infected samples is shown in Fig. 2D. A list of DEmiRNAs identified in this study is provided in Supplementary Table S1.

### Enrichment analysis of the DEmiRNAs during *T. gondii* infection

**Fig. 3:**
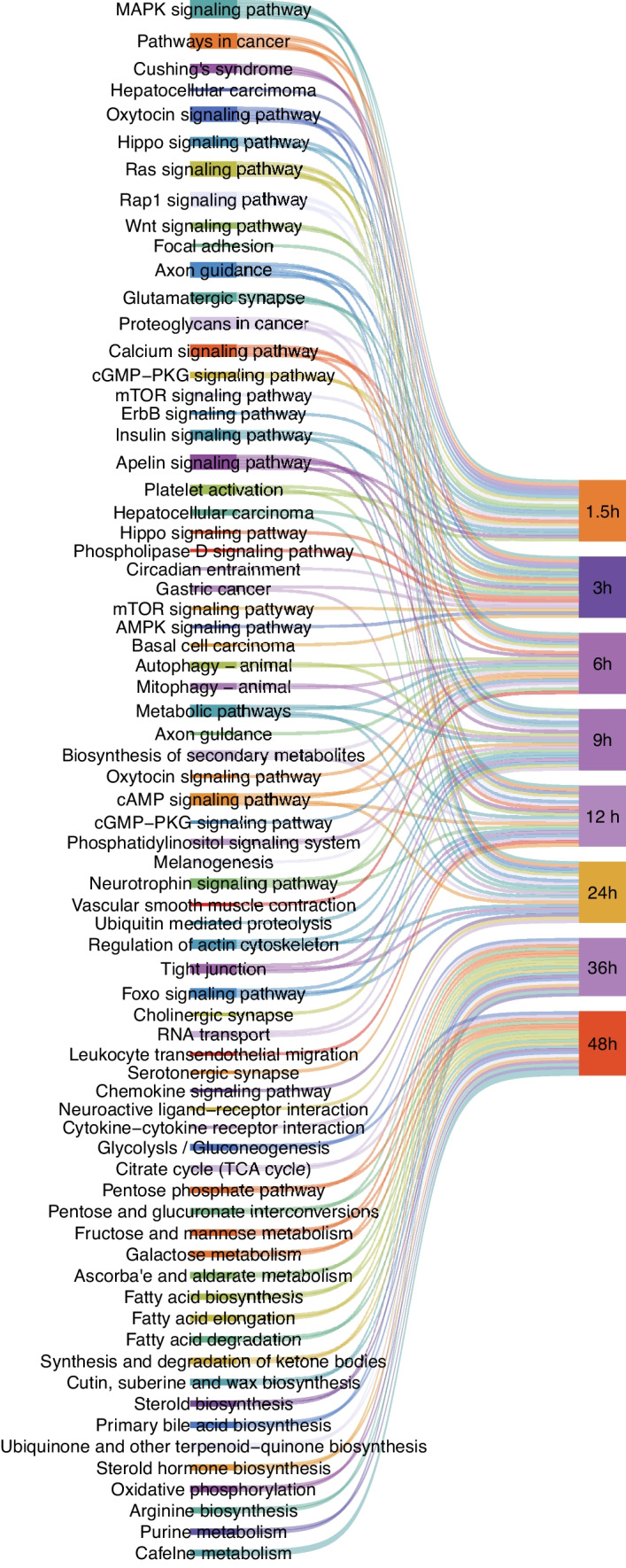
KEGG pathway analysis of the DEmiRNA host genes in HFF cells at different times after *T. gondii* infection. Sankey diagram shows the top 20 predominant pathways at 1.5 hpi, 3 hpi, 6 hpi, 9 hpi, 12 hpi, 24 hpi, 36 hpi, and 48 hpi, respectively

There was a clear difference in the number of DEmiRNAs at different time points after *T. gondii* infection, which reflects temporal differences in the host response to *T. gondii* infection. The DEmiRNAs were significantly enriched in biological process (Supplementary Fig. 1), and most DEmiRNAs were enriched in the regulation of RNA polymerase II (GO:0045944), cell differentiation (GO:0030154), multicellular organism development (GO:0007275), apoptotic process (GO:0006915), and phosphorylation (GO:0016310). The KEGG pathway enrichment was used to analyze the significantly enriched pathways of the DEmiRNAs. The top 20 pathways are shown in Fig. 3. Most DEmiRNAs were involved in metabolic-related pathways, such as metabolic pathways (ko01100), glycolysis/gluconeogenesis (ko00010), and citrate cycle (TCA cycle) (ko00020).

### Identification of DEcircRNAs

**Fig. 4:**
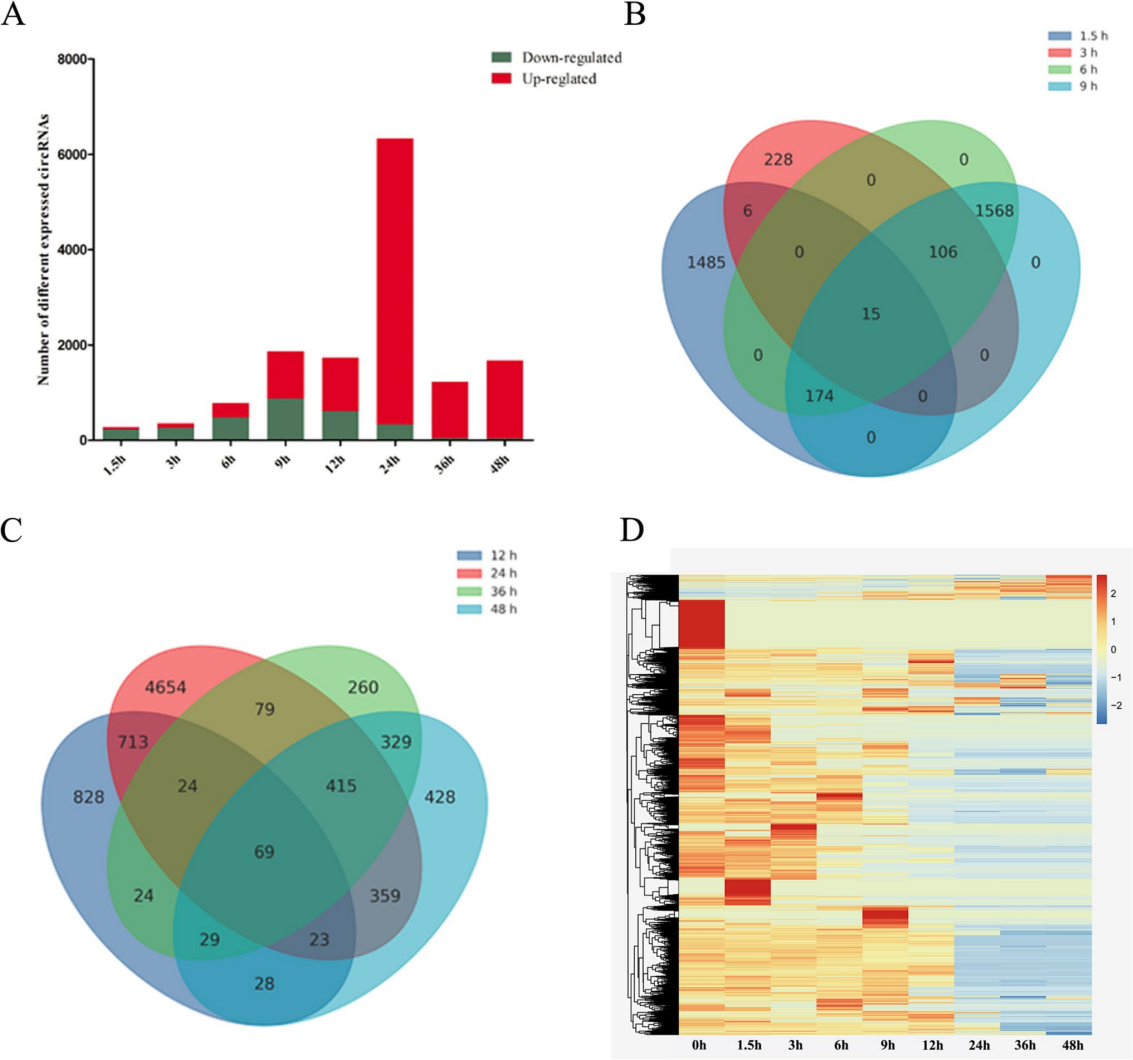
Global changes in circRNA expression after *T. gondii* infection. (**A**) DEcircRNAs detected in HFF cells at different time points after *T. gondii* infection. The x-axis shows the time points after *T. gondii* infection and the y-axis shows the number of DEcircRNAs. Red and green colors represent the upregulated and downregulated DEcircRNAs, respectively. Four-ellipse Venn diagram outlining the common and unique DEcircRNAs at 1.5 to 9 hpi (**B**) and 12 to 48 hpi (**C**). Hierarchical clustering heatmap of the DEcircRNAs at different time points after *T. gondii* infection. Clustering was performed as log_10_ (FPKM + 1) values. The values of circRNA expression are indicated by the color scale; from red to blue indicating highly expressed and low expressed circRNAs, respectively. Each column represents a time point after infection, and each row represents one circRNA (**D**)

Our analysis identified 276 DEcircRNAs (44 upregulated and 232 downregulated), 355 DEcircRNAs (95 upregulated and 260 downregulated), 782 DEcircRNAs (302 upregulated and 480 downregulated), 1863 DEcircRNAs (986 upregulated and 877 downregulated), 1738 DEcircRNAs (1122 upregulated and 616 downregulated), 6336 DEcircRNAs (6004 upregulated and 332 downregulated), 1229 DEcircRNAs (1187 upregulated and 42 downregulated), and 1680 DEcircRNAs (1642 upregulated and 38 downregulated) at 1.5, 3, 6, 9, 12, 24, 36, and 48 hpi, respectively (Fig. 4A). Eight DEcircRNAs (hsa-circ_0004662, hsa-circ_0005472, hsa-circ_0039467, hsa-circ_0083081, hsa-circ_0083618, hsa-circ_0115893, hsa-circ_0133744, and hsa-circ_0133745) were co-expressed at different time points. Fifteen DEcircRNAs were co-expressed during 1.5 to 9 hpi (Fig. 4B) and 69 DEcircRNAs were co-expressed during 12 to 48 hpi (Fig. 4C). The hierarchical clustering of miRNA expression profiles between control and infected samples is shown in Fig. 4D. A list of the DEcircRNAs is provided in Supplementary Table S2.

### Functions of DEcircRNA host genes

**Fig. 5:**
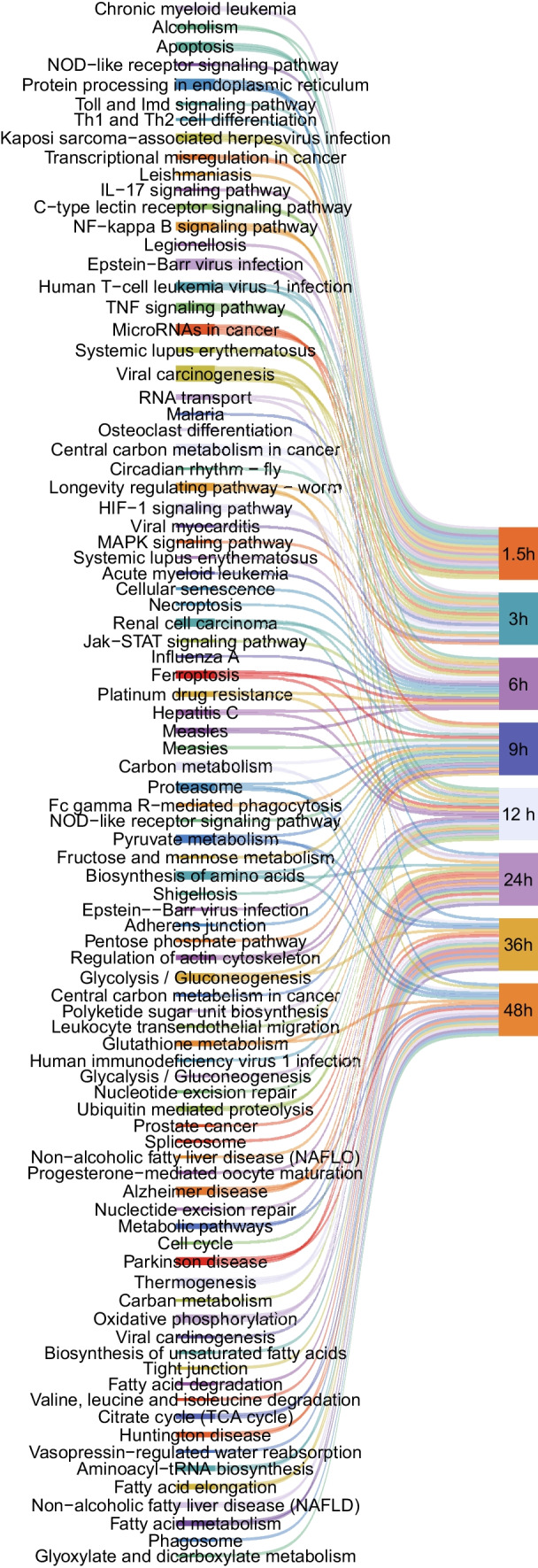
KEGG pathway analysis of the DEcircRNA host genes in HFF cells at different times after *T. gondii* infection. Sankey diagram shows the top 20 predominant pathways at 1.5 hpi, 3 hpi, 6 hpi, 9 hpi, 12 hpi, 24 hpi, 36 hpi, and 48 hpi, respectively

GO enrichment analysis was performed to elucidate the biological function of the target host genes of DEcircRNAs and their role in *T. gondii* infection. According to GO functional annotation, the DEcircRNA host genes were classified into three categories: biological process, cellular component, and molecular function. The GO terms are shown in Supplementary Fig. 2. The DEcircRNAs were most significantly enriched in the biological process category, and most DEcircRNAs were enriched in cellular process (GO:0009987), metabolic process (GO:0008152), biological regulation (GO:0065007), regulation of biological process (GO:0050789), and response to stimulus (GO:0050896). The KEGG pathway enrichment was used to analyze the significantly enriched pathways of the DEcircRNAs. The top 20 pathways are shown in Fig. 5. Most DEcircRNAs were involved in immune-related signaling pathways, such as NOD-like receptor signaling pathway, apoptosis, TNF signaling pathway, and metabolic-related pathway, such as TCA cycle pathway and glutathione metabolism.

### Validation of the deep sequencing results by qRT-PCR

**Fig. 6:**
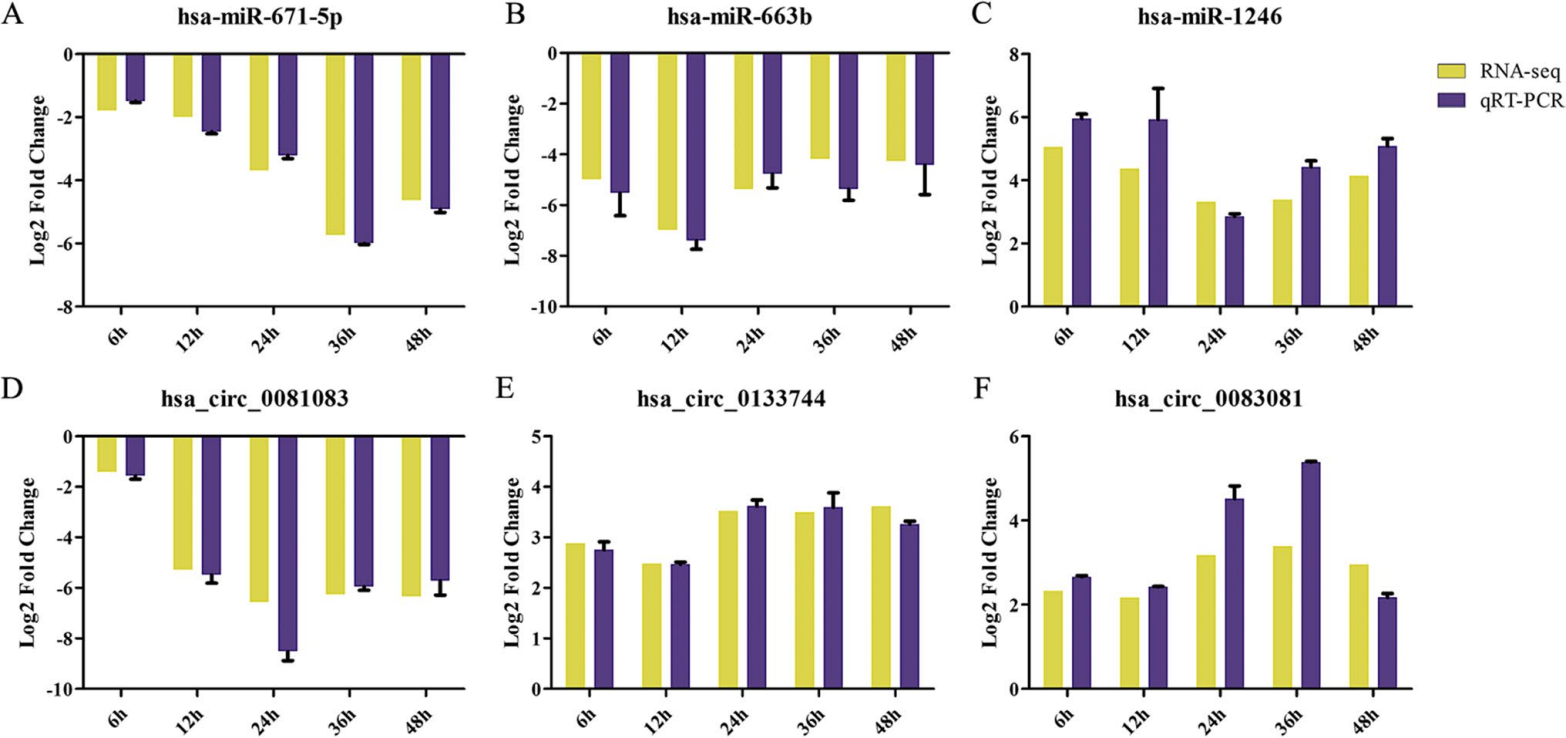
A qRT-PCR-based validation of the expression of representative EDmiRNAs (hsa-miR-671-5p, hsa-miR-663b, and hsa-miR-1246) (**A**–**C**) and DEcircRNAs (hsa-circ_0081083, hsa-circ_0133744, and hsa-circ_0083081) (**D**–**F**). The x-axis shows the time points after *T. gondii* infection and the y-axis shows the relative expression levels. The *U6* gene and *GAPDH* gene were used for miRNA and circRNA normalization, respectively

To confirm the differential gene expression obtained from the transcriptome sequencing data, a subset of unigenes co-expressed during *T. gondii* infection were randomly selected for qRT-PCR verification. These included three DEmiRNAs (hsa-miR-671-5p, hsa-miR-663b, and hsa-miR-1246) (Fig. 6A–C) and three DEcircRNAs (hsa-circ_0081083, hsa-circ_0133744, and hsa-circ_0083081) (Fig. 6D–F). The results obtained by qRT-PCR analysis were consistent with that of the high-throughput sequencing, suggesting the reliability and accuracy of the RNA-seq-based transcriptomic analysis.

### Analysis of DEcircRNAs-DEmiRNAs interaction

**Fig. 7:**
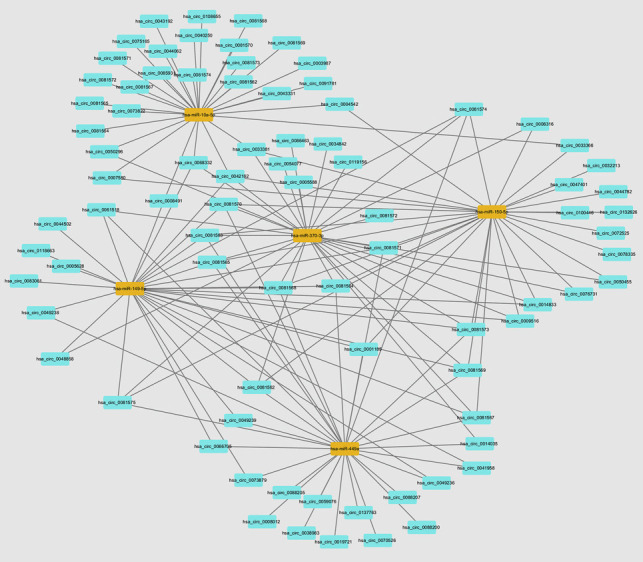
The competing endogenous RNA (ceRNA) network of miRNAs and circRNAs in HFF cells infected by *T. gondii*. Cyan rectangles represent the circRNAs and orange nodes represent the miRNAs

CircRNAs can act as miRNA decoys or sponges to regulate host gene expression. To reveal correlations between DEmiRNAs and DEcircRNAs and elucidate the possible role of miRNAs and circRNAs in *T. gondii* infection, the ceRNA network was constructed. In the ceRNA network, one miRNA appears to be associated with one or more upstream circRNAs (Fig. 7). These circRNAs could play a role as biological targets of *T. gondii* infection.

## Discussion

In this study, we used sequencing technology to investigate the dynamic and differential expression profiles of miRNAs and circRNAs in HFF cells in the presence or absence of *T. gondii* infection at 0, 1.5, 3, 6, 9, 12, 24, 36, and 48 hpi. Our aim was to identify transcripts with an essential role in the host response to *T. gondii* during the parasite infection lytic cycle. Our study showed that *T. gondii* infection caused significant alterations in the expression of circRNAs involved in immune-related pathways, such as NOD-like receptor signaling pathway, apoptosis, and NF-kappa B signaling pathway at the early stage of infection. These results are consistent with previous transcriptomics studies showing that *T. gondii* infection deregulates many genes involved in immune regulation and activation (Hunter and Sibley 2012; Liu et al. 2018; Wang et al. 2018).

Apoptosis is a form of programmed cell death that can limit the proliferation of intracellular pathogens by depriving them of their intracellular niche (Wallach and Kang 2018). To counter apoptosis, *T. gondii* disrupts the mitochondrial and death receptor-mediated apoptotic pathway (Vutova et al. 2007; Wallach and Kang 2018) and inhibits the expression of caspases-8, -9, -3, and -7 in different cell lines (Hwang et al. 2010; Graumann et al. 2015; Afshar-Khamseh et al. 2021). *T. gondii* can delay apoptosis of infected human neutrophils by deactivating apoptotic caspases via activating cytosolic proliferating cell nuclear antigen (Wallach and Kang 2018). In the present study, we detected an increase in the expression of miR-142a-3p which plays a role in immune regulation and apoptosis in many cells by targeting p53, TNFAIP2, and GLUT3 (Lu et al. 2015). These results add more evidence to the literature showing the ability of *T. gondii* to evade and manipulate host immune response to establish an optimal replicative niche (Seeber and Steinfelder 2016; Lima et al. 2021).

*T. gondii* infection is involved in several neuropsychiatric disorders (Elsheikha and Zhu 2016; Elsheikha et al. 2016; Liang et al. 2021). However, the mechanisms underlying this connection remain inconclusive. Interestingly, our study revealed that *T. gondii* can activate DEmiRNAs associated with nervous system-related pathways, such as axon guidance (ko04360), neurotrophin signaling pathway (ko04722), and neuroactive ligand-receptor interaction (ko04080). We also detected a decreasing expression trend of miRNA-146a-5p during *T. gondii* infection, suggesting that *T. gondii* may contribute to brain dysfunction via altering miRNAs expression. This agrees with the literature reporting the involvement of miRNAs in the pathogenesis of diseases of the nervous system (Hutchison et al. 2009). The miRNA-146a can switch microglial phenotypes to resist the pathological processes and cognitive degradation associated with Alzheimer’s disease (MacRae et al. 2012).

Previous studies revealed how the metabolism of *T. gondii* and the host is altered after infection (Blume et al. 2009, 2015; Ma et al. 2019, 2020, 2021; Olson et al. 2020). In the present study, we have shown that *T. gondii* infection changes the host cells’ metabolism by altering the expression of circRNAs involved in TCA cycle, metabolic pathways, fatty acid metabolism, glutathione metabolism, and glycolysis/gluconeogenesis. The TCA cycle is essential for *T. gondii* growth (Blume et al. 2009). In the absence of glucose or under genetically ablated glycolysis, *T. gondii* can utilize an intrinsic protein, gluconeogenic enzyme fructose bisphosphatase 2, to perform glutaminolysis and generate carbon for gluconeogenesis (Blume et al. 2009, 2015). The exact impact of miRNAs and circRNAs on the regulation of host metabolism during *T. gondii* infection merit further investigated.

CircRNAs act as miRNA decoys or sponges to influence miRNA function and regulate the expression of target mRNAs (Panda 2018). To better understand the regulatory effects of DEcircRNA, we predicted the putative circRNA-miRNA interaction networks based on the DEcircRNAs and DEmiRNAs. The results showed that DEmiRNAs (hsa-miR-149-5p, hsa-miR-370-3p, hsa-miR-449a, and hsa-miR-150-5p) were regulated by DEcircRNAs (hsa-circ_0081574, hsa-circ_0081567, hsa-circ_0081569, and hsa-circ_0081575). The miR-149-5p serves as a negative regulator of inflammatory and immune responses (Hübner et al. 2020; Li et al. 2023) and regulates the transcription levels of IL-10, TREM-1, and PIM1, which have an anti-inflammatory activity (Neamah et al. 2019; Wang et al. 2023; Yue et al. 2023), highlighting the role of the identified DEcircRNAs in regulating the immune-inflammatory response during *T. gondii* infection.

## Conclusion

This study revealed changes in the transcriptional patterns of miRNAs and circRNAs in HFF cells during *T. gondii* lytic cycle. Many DEmiRNAs and DEcircRNA were identified at different time points post *T. gondii* infection. The DEmiRNAs and DEcircRNAs were mainly involved in key physiopathological processes, including apoptosis, metabolism, signal transduction, and immune responses. Our data adds more insight into the role of miRNAs and circRNAs in the pathogenesis of *T. gondii* infection. The mechanism by which these DEmiRNAs and DEcircRNAs regulate antiparasitic responses requires further investigation.

### Supplementary Information

Below is the link to the electronic supplementary material.

Supplementary Fig. 1 Functional enrichment analysis of the predicted target genes of the differentially expressed miRNAs detected in HFF cells after *T. gondii* infection. Sankey plot shows the most significantly enriched GO terms in the biological process at 1.5 hpi, 3 hpi, 6 hpi, 9 hpi, 12 hpi, 24 hpi, 36 hpi, and 48 hpi (PDF 434 KB)

Supplementary Fig. 2 Functional enrichment analysis of the predicted target genes of the differentially expressed circRNAs detected in HFF cells after *T. gondii* infection. Sankey plot shows the most enriched GO terms at 1.5 hpi, 3 hpi, 6 hpi, 9 hpi, 12 hpi, 24 hpi, 36 hpi, and 48 hpi (PDF 1.15 MB)

Table S1 A list of all differentially expressed miRNAs detected in HFF cells at different time points after *T. gondii* infection (XLS 99.5 KB)

Table S2 A list of all differentially expressed circRNAs detected in HFF cells at different time points after *T. gondii* infection (XLS 3.46 MB)

## Data Availability

The RNA-seq data presented in this study were deposited in the NCBI repository under accession numbers PRJNA721229 and PRJNA769531.
