# Supplementary figures and images for: Expression profiles of host miRNAs and circRNAs and ceRNA network during Toxoplasma gondii lytic cycle
Source: Parasitol Res. 2024 Feb 29;123(2):145. doi: 10.1007/s00436-024-08152-x (PMC10902104; doi:10.1007/s00436-024-08152-x)

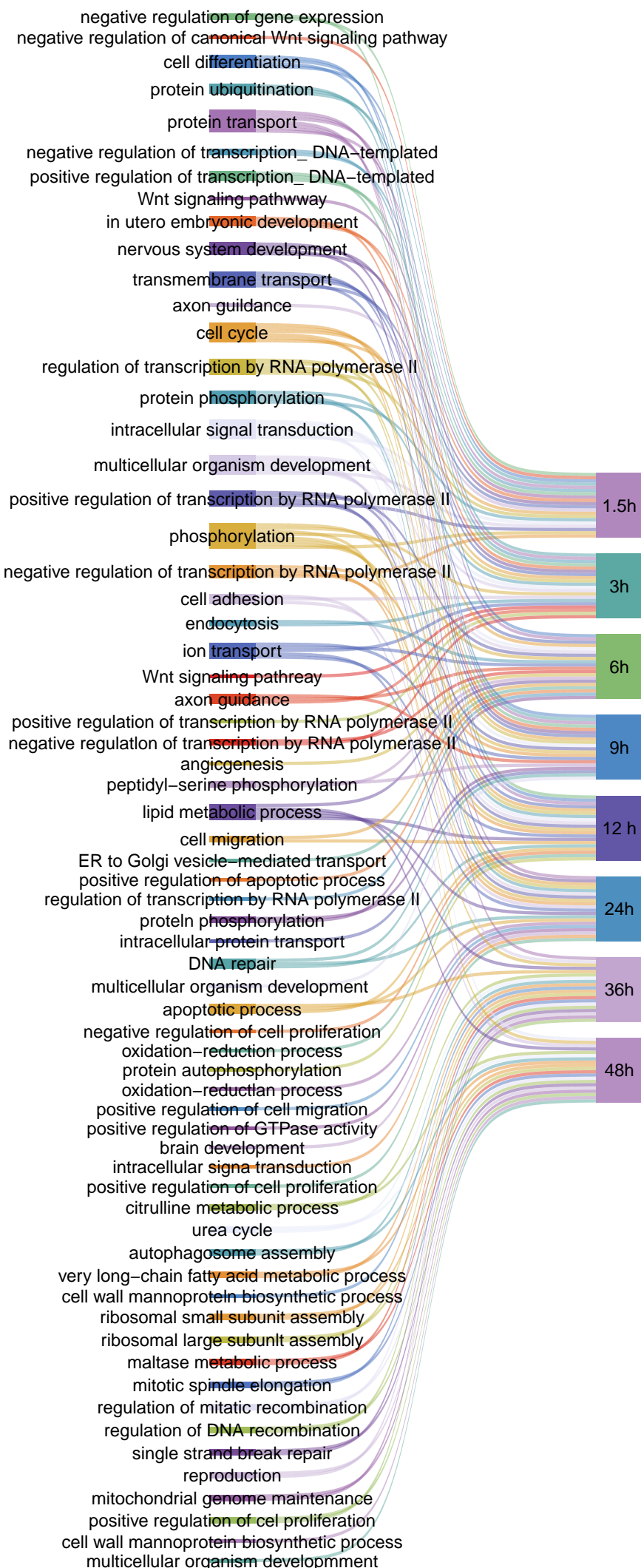

Supplement: Supplementary file 1 — Functional enrichment analysis of the predicted target genes of the differentially expressed miRNAs detected in HFF cells after T. gondii infection. Sankey plot shows the most significantly enriched GO terms in the biological process at 1.5 hpi, 3 hpi, 6 hpi, 9 hpi, 12 hpi, 24 hpi, 36 hpi, and 48 hpi (PDF 434 KB) [file 436_2024_8152_MOESM1_ESM.pdf]

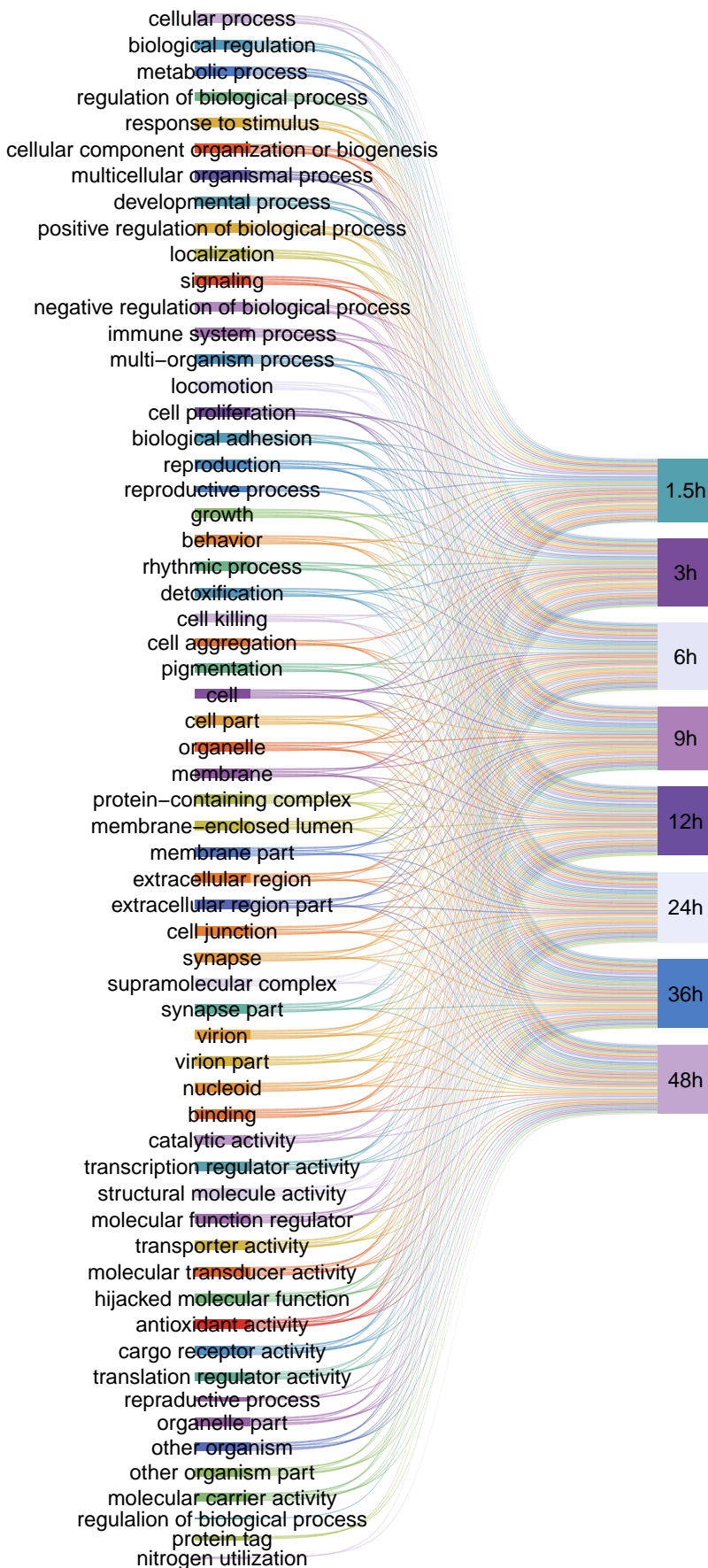

Supplement: Supplementary file 2 — Functional enrichment analysis of the predicted target genes of the differentially expressed circRNAs detected in HFF cells after T. gondii infection. Sankey plot shows the most enriched GO terms at 1.5 hpi, 3 hpi, 6 hpi, 9 hpi, 12 hpi, 24 hpi, 36 hpi, and 48 hpi (PDF 1.15 MB) [file 436_2024_8152_MOESM2_ESM.pdf]
